# Aminoglycoside use and intensive care unit-acquired weakness: A systematic review and meta-analysis

**DOI:** 10.1371/journal.pone.0230181

**Published:** 2020-03-19

**Authors:** Tao Yang, Zhi-Qiang Li, Hong-Liang Li, Jian-Xin Zhou, Guang-Qiang Chen

**Affiliations:** 1 Department of Critical Care Medicine, Beijing Tiantan Hospital, Capital Medical University, Beijing, China; 2 Department of Critical Care Medicine, North China University of Science and Technology Affiliated Hospital, Tangshan, China; University of British Columbia, CANADA

## Abstract

**Background:**

The relationship between aminoglycoside use and intensive care unit (ICU)-acquired weakness remains controversial. In the present study, we performed a systematic review and meta-analysis to examine the relationship between aminoglycoside use and ICU-acquired weakness in critically ill patients.

**Methods:**

The PubMed, Embase, Web of Science, Cochrane Central Register of Controlled Trials and Cumulative Index of Nursing and Allied Health Literature databases were searched from the earliest available date to July 10, 2019. Randomized controlled trials and prospective cohort studies examining the relationship between aminoglycosides and ICU-acquired weakness in adult ICU patients were included. Two authors independently screened titles/abstracts, reviewed full text and extracted data from the included studies. We performed the Meta-analysis using Stata version 15.0 and used the DerSimonian-Laird random effects model for data analyses. Heterogeneity was evaluated using the χ^2^ statistic and *I*^2^ statistic. Publication bias was evaluated with funnel plots qualitatively, the Begg’s test and Egger’s test quantitatively.

**Results:**

Ten prospective cohort studies were included and analysed in this review. The overall effect sizes of the studies revealed a statistically significant relationship between aminoglycoside use and ICU-acquired weakness (OR, 2.06; 95%CI, 1.33–3.21; *I*^*2*^ = 56%). Subgroup and sensitivity analyses suggested a significant association between aminoglycoside use and studies limited to patients with clinical weakness (OR, 2.74; 95%CI, 1.83–4.10; *I*^2^ = 0%), and not to studies limited to patients with abnormal electrophysiology (OR, 1.78; 95%CI, 0.94–3.39; *I*^*2*^ = 59%), a large sample size (OR, 1.81; 95%CI, 0.97–3.39; *I*^2^ = 75%), or low risk of bias (OR, 1.59; 95%CI, 0.97–2.60; *I*^2^ = 56%); however, statistical heterogeneity was obvious. There were no significant publication biases found in the review.

**Conclusions:**

The review revealed a significant relationship between aminoglycoside use and ICU-acquired weakness.

## Introduction

Intensive care unit-acquired weakness (ICUAW) is an acute neuromuscular impairment of critically ill patients. ICUAW is associated with prolonged weaning from mechanical ventilation, increased healthcare-related costs, longer intensive care unit (ICU) and hospital stays, and higher ICU- and hospitalization-related mortality[1–9]. Because of strong bactericidal activity and low rates of resistance, aminoglycosides therapy is recommended for treatment of life-threatening infections in critically ill patients[10, 11]. Aminoglycosides are still used for certain difficult-to-treat infections in many ICUs, and are alternate antimicrobials in cases of antibacterial resistance[12]. Neuromuscular blockade was reported to be a rare but potentially dangerous side effect of aminoglycosides[12]. ICUAW occurs frequently in critically ill patients, but the relationship between aminoglycoside therapy and ICUAW remains unclear. Researchers have raised significant concerns about the role of aminoglycoside therapy in ICUAW development and have attempted to examine the association. Some clinical trials[6, 7, 13–15] have indicated that a statistically significant side effect of aminoglycosides in developing ICUAW, yet others[16–20] have not. In this review, we performed a systematic review and meta-analysis of randomized controlled trials (RCTs) and prospective cohort studies to assess the relationship between aminoglycoside use and ICUAW development.

No universal consensus or recommendation on the definition or classification of the disease exists; after consulting the literature[21], the relatively broad term “intensive care unit-acquired weakness (ICUAW)” was selected for use in this review. Three diagnostic methods were recommended to identify ICUAW[21, 22]: manual muscle testing (Medical Research Council (MRC) weakness scale), electrophysiological studies, and the histopathology of muscle or nerve tissue. However, muscle or nerve tissue biopsy was rarely used in the studies. This review explores the adverse effect of aminoglycosides on ICUAW development, from patients with clinical weakness to patients with clinically undetectable neuromuscular dysfunction.

## Materials and methods

This study was performed following the Preferred Reporting Items for Systematic Reviews and Meta-Analyses: the PRISMA statement[23].

### Search strategy

The following databases were searched for the pertinent English language studies from inception through July 10, 2019: PubMed, Embase, Web of Science, Cochrane Central Register of Controlled Trials, and Cumulative Index of Nursing and Allied Health Literature. We used the search terms for PubMed (S1 Text) and the other databases. Additionally, we performed a manual search of references cited by the included articles and relevant review articles to identify other eligible studies.

### Selection criteria

The inclusion criteria were as follows: age > 18; RCTs and prospective cohort studies; diagnoses of ICUAW made using diagnostic tests (electrophysiological studies, histopathology of muscle or nerve tissue) or manual muscle testing (Medical Research Council (MRC) weakness scale); and studies that evaluated the use of aminoglycosides and incidence of ICUAW. The exclusion criteria were as follows: patients with primary polyneuropathies (e.g., Guillain-Barré syndrome, myasthenia gravis) or myopathies (e.g., idiopathic inflammatory myopathies); and studies with insufficient data reported.

### Study selection and data abstraction

Studies were independently reviewed and selected by two reviewers (T.Y. and Z.Q.L.) based on the inclusion criteria. Two reviewers (T.Y. and Z.Q.L.) independently extracted the following data from each study using a standardized data collection form: author information, publication year, study design, study location, inclusion and exclusion criteria, tools of neuromuscular evaluation, number of participants, ICUAW incidence, number of ICUAW patients who were given and not given aminoglycosides and total number of patients given and not given aminoglycosides. Unadjusted event rates of ICUAW were calculated by dividing the number of patients with ICUAW who were given aminoglycosides by the total number given aminoglycosides. Disagreements in study selection or data extraction were resolved by either consensus or a third-party decision.

### Study quality assessment

The methodological quality of each study was independently assessed by two reviewers (T.Y. and Z.Q.L.) using the Newcastle–Ottawa scale[24]

### Data analysis

We performed the meta-analysis using Stata version 15.0 (StataCorp, College Station, TX, USA), analyzed the results using odds ratios (ORs) and 95% confidence intervals (CIs). We used the DerSimonian and Laird random effects model for data analyses. We assessed the heterogeneity using the χ^2^ statistic with P ≤ 0.1 considered statistically significant. We estimated the impact of statistical heterogeneity on the study results by calculating the *I*^2^ statistic. Values of the *I*^2^ statistic above 50% were regarded as a cutoff point for considerable heterogeneity. Subgroup and sensitivity analyses examined (1) studies using clinical muscle testing and electrophysiology as a diagnostic method; (2) studies with relatively large sample sizes (exclusion of studies with a sample size less than 100); (3) studies with low risk of bias (exclusion of studies with Newcastle–Ottawa scale score < 7). We examined the publication bias using Egger’s linear regression test and Begg’s rank correlation test for quantitative assessment, using funnel plots for qualitative assessment.

## Results

### Study search and selection

**Fig 1:**
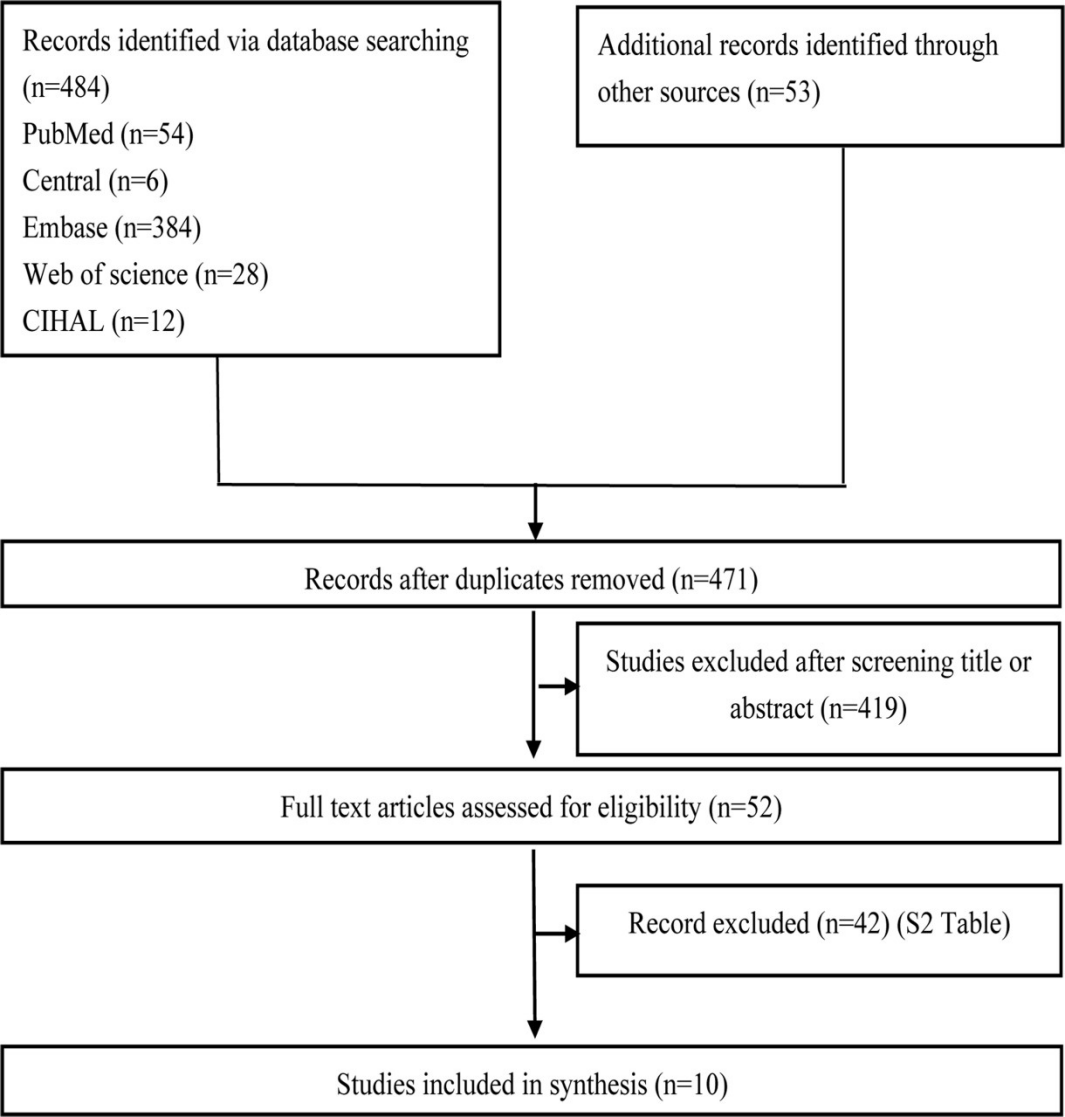


The initial search yielded 484 citations (Fig 1). Fifty-three additional articles were yielded after further review of the bibliographies. After screening titles and abstracts, fifty-two articles were selected for full-text review. Forty-two articles were excluded (S1 Table), and 10 studies[6, 7, 13–20] were finally included in this review.

### Study characteristics and quality

Characteristics of included studies in this systematic review are presented in Table 1. There were 10 prospective cohort studies[6, 7, 13–20] included in the review. The studies included 1363 patients. The studies were carried out in the Netherlands[13, 14], Greece[7, 16], Germany[15, 17], Belgium[18], Spain[19], France[6]. Seven studies[14–20] evaluated ICUAW using electrophysiological studies, and three studies[6, 7, 13] evaluated ICUAW using the MRC scale. Mortality rates for patients with ICUAW ranged from 17%[6] to 66%[20]. Of the 2 included studies[7, 13], patients with ICUAW had a significantly higher ICU mortality rate compared with the patients who did not develop ICUAW (P < 0.05).

**Table 1:** Characteristics of selected studies.

Study	Study Design	Country	Setting	Population	n	Examination	ICUAW ^a^	AM/non-AM	ICU Mortality (%)^a^
Wieske et al, 2014[13]	Prospective cohort	Netherlands	MSICU	MV ≥ 2 d	212	Clinical	51 vs 52	81 vs 131	34% vs 9%
Anastasopoulos et al, 2011[16]	Prospective cohort	Greece	MSICU	ICU-LOS ≥ 7 d	190	EMG	20 vs 20	72 vs 118	32.5% vs NR
Weber-Carstens et al, 2010[17]	Prospective cohort	Germany	SICU	MV and SAPS-Ⅱ≥ 20	40	EMG	6 vs 16	9 vs 31	NR
Nanas et al, 2008[7]	Prospective cohort	Greece	MSICU	LOS > 10 d	185	Clinical	28 vs 16	80 vs 105	36% vs 20%
Hermans et al, 2007[18]	Prospective cohort	Belgium	MICU	MV > 7 d	412	EMG	24 vs 164	59 vs 353	NR
Amaya-Villar et al, 2005[19]	Prospective cohort	Spain	MSICU	COPD, MV > 48 h, high-dose steroids	26	EMG	2 vs 7	2 vs 24	33.3% vs 17.6%
De Jonghe et al, 2002[6]	Prospective cohort	France	MICU, SICU	MV > 7 d and awake	95	Clinical	16 vs 8	46 vs 49	17% vs 6%
De Letter et al, 2001[14]	Prospective cohort	Netherlands	MSICU	MV ≥ 4 d	97	EMG	19 vs 15	37 vs 60	NR
Garnacho-Montero et al, 2001[20]	Prospective cohort	Spain	MSICU	MV > 10 d and sepsis with MOF	73	EMG	21 vs 29	31 vs 42	66% vs 52%
Mohr et al, 1997[15]	Prospective cohort	Germany	MSICU	MOF ≥5 d	33	EMG	7 vs 0	16 vs 17	NR

ICUAW, intensive care unit-acquired weakness; AM, aminoglycosides; ICU, intensive care unit; MSICU, medical surgical ICU; MV, mechanical ventilation; LOS, length of stay; EMG, Electromyography; NR, not reported; SICU, surgical ICU; SAPS, simplified acute physiology score; MICU, medical ICU; COPD, chronic obstructive pulmonary disease; MOF, multiple organ failure.

^a^ comparison between AM and non-AM.

The methodological quality assessment of the included reports is presented in Table 2. The risk of bias of the prospective observational cohort studies was obvious in general. One study[18] made statistical comparisons with multivariable regression analysis for aminoglycosides, and therefore the other nine studies received no scores for comparability. Four studies[7, 14, 15, 19] did not report whether the assessments were independently blinded for physical therapists or clinicians.

**Table 2:** Methodology and reporting assessment.

Newcastle-Ottawa quality assessment scale for cohort studies									
Studies	Selection				Comparability	Outcome			Score
	Exposed representative?	Non-exposed representative?	Ascertainment of exposure	Outcome of interest not present at start		Assessment of outcome	Adequate duration of follow-up	Completeness of follow-up	
		
Wieske et al, 2014[13]	Y	Y	Y	Y	N, N	Y	Y	Y	7
Anastasopoulos et al, 2011[16]	Y	Y	Y	Y	N, N	Y	Y	Y	7
Weber-Carstens et al, 2010[17]	Y	Y	Y	Y	N, N	Y	Y	Y	7
Nanas et al, 2008[7]	Y	Y	Y	Y	N, N	N	Y	Y	6
Hermans et al, 2007[18]	Y	Y	Y	Y	Y, Y	Y	Y	Y	9
Amaya-Villar et al, 2005[19]	N	Y	Y	Y	N, N	N	Y	Y	5
De Jonghe et al, 2002[6]	Y	Y	Y	Y	N, N	Y	Y	Y	7
De Letter et al, 2001[14]	Y	Y	Y	Y	N, N	N	Y	Y	6
Garnacho-Montero et al, 2001[20]	Y	Y	Y	Y	N, N	Y	Y	Y	7
Mohr et al, 1997[15]	N	Y	Y	N	N, N	N	Y	Y	4

Y—criteria satisfied, N—criteria not satisfied

### Aminoglycosides and ICUAW

**Fig 2:**
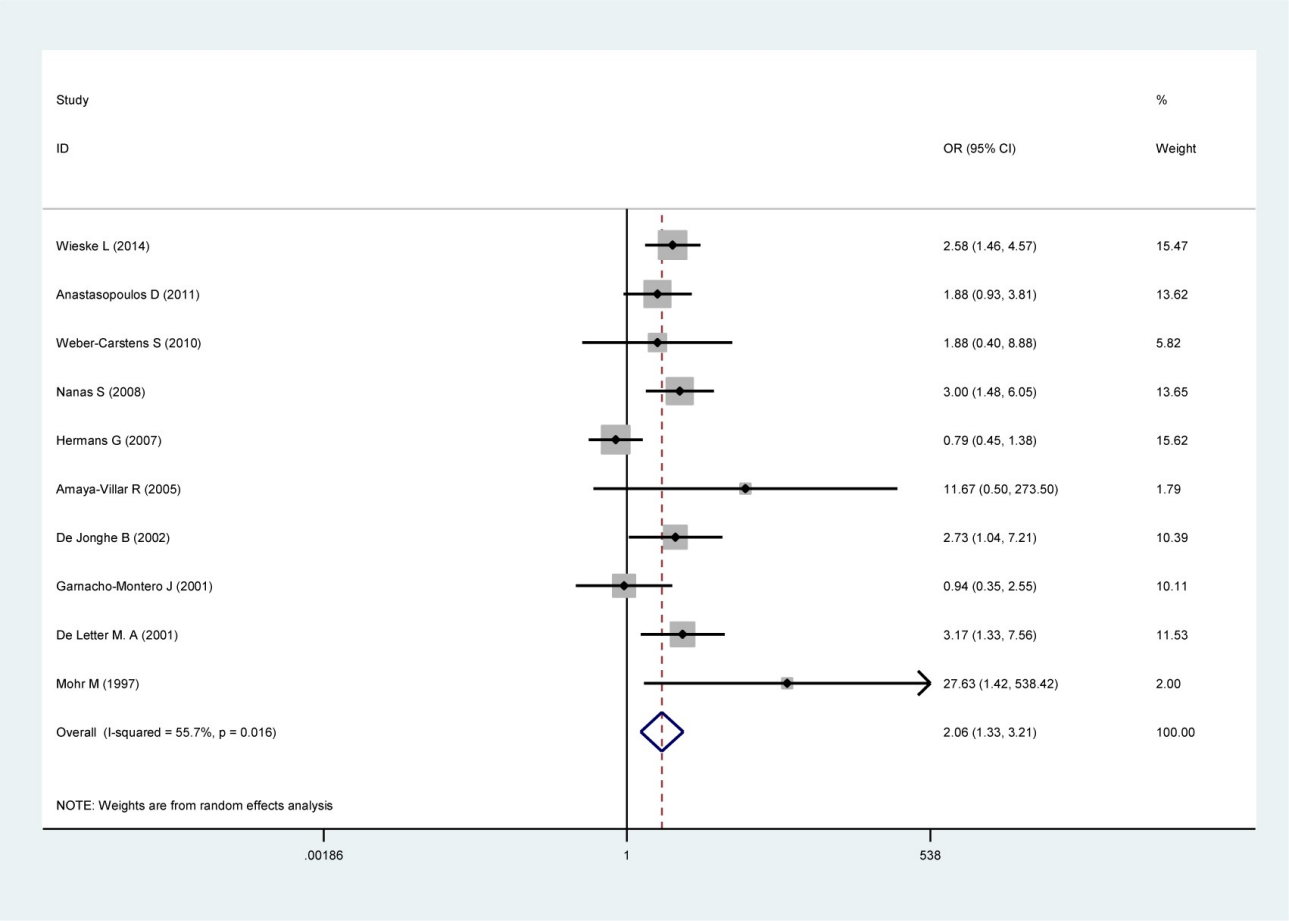


When the 10 studies were pooled together (Fig 2), the effect size analysis demonstrated that the use of aminoglycosides was significantly associated with ICUAW (OR 2.06; 95% CI 1.33–3.21; P < 0.01). Data were pooled using a random effects model considering the observed heterogeneity (τ^2^ = 0.24; χ^2^ = 20.31, df = 9 (P = 0.016); *I*^2^ = 55.7%). The overall incidence of ICUAW was 45% in the aminoglycoside group versus 35% in the control group.

### Subgroup analyses

#### 1. Clinical assessment versus electrophysiology

The subgroup analyses are presented in Table 3. Three studies[6, 7, 13] examined an association between the use of aminoglycosides and patients with clinical weakness and showed an event rate of 46% in the aminoglycoside group and 27% in the control group. The pooled effect size (OR 2.74; 95%CI 1.83–4.10; P < 0.01) revealed a significant association with a random effects model considering the heterogeneity (τ^2^ = 0; χ^2^ = 0.1, df = 2 (P = 0.95); *I*^2^ = 0%). Seven studies[14–20] evaluated the association between the use of aminoglycoside and patients with abnormal electrophysiology and indicated an incidence of 44% in the aminoglycoside group versus 39% in the control group. The overall effect size (OR 1.78; 95% CI 0.94–3.39; P = 0.08) demonstrated no significant association. Data were pooled using a random effects model considering the observed heterogeneity (τ^2^ = 0.36; χ^2^ = 14.42, df = 6 (P = 0.025); *I*^2^ = 58.4%). No statistically significant heterogeneity between the subgroups was found based on a test of the interaction (P = 0.27).

**Table 3:** Subgroup and sensitivity analyses.

Analyses	Study	n	*I*^2^(%)	Ph	OR	95%CI	Pe	Pi	Incidence aminoglycoside	Incidence control
Primary analysis	[6, 7, 13–20]	1363	56%	0.02	2.06	1.33–3.21	<0.01		45%	35%
Diagnostic method										
Clinical assessment	[6, 7, 13]	492	0%	0.95	2.74	1.83–4.10	<0.01		46%	27%
Electrophysiology	[14–20]	871	59%	0.02	1.78	0.94–3.39	0.08	0.27	44%	39%
Sample size										
n≥100	[7, 13, 16, 18]	999	75%	<0.01	1.81	0.97–3.39	0.06		42%	36%
n<100	[6, 14, 15, 17, 19, 20]	364	34%	0.18	2.47	1.26–4.83	<0.01	0.51	50%	34%
Sensitivity analysis	[6, 13, 16–18, 20]	1022	56%	0.05	1.59	0.97–2.60	0.06		46%	40%

*I*^2^, *I*-squared statistic test for heterogeneity; Ph, P value for test of heterogeneity; OR, odds ratio; CI, confidence intervals; Pe, P value for the effect estimate for each subgroup; Pi, P value for interaction tests of heterogeneity between subgroups.

#### 2. Sample sizes (n ≥ 100 versus n < 100)

After incorporating the results of the four studies[7, 13, 16, 18] with sample sizes more than 100, the pooled effect size (OR 1.81; 95% CI 0.97–3.39; P = 0.06) did not reveal a significant association between aminoglycoside use and ICUAW with a random effects model considering the observed heterogeneity (τ^2^ = 0.30; χ^2^ = 11.81, df = 3 (P < 0.01); *I*^2^ = 74.6%), with an incidence of 42% in the aminoglycoside group and 36% in the control group. The remaining six studies[6, 14, 15, 17, 19, 20] with relatively small sample sizes (n < 100) demonstrated an unadjusted incidence in the aminoglycoside group of 50% versus 34% in the control group. The pooled effect size (OR 2.47; 95% CI 1.26–4.83; P < 0.01) showed a significant association with a random effects model considering the observed heterogeneity (τ^2^ = 0.22; χ^2^ = 7.49, df = 5 (P < 0.186); *I*^2^ = 33.3%). No statistically significant heterogeneity between the subgroups was found based on a test of the interaction (P = 0.51).

### Sensitivity analysis

The sensitivity analysis was shown in Table 3. After excluding studies[7, 14, 15, 19] with high risk of bias, the included studies[6, 13, 16–18, 20] did not reveal a significant association between aminoglycoside use and ICUAW (OR 1.59; 95% CI 0.97–2.60; P = 0.06) with a random effects model considering the observed heterogeneity (τ^2^ = 0.20; χ^2^ = 11.26, df = 5 (P = 0.05); *I*^2^ = 56%). Incidence of ICUAW were 46% in the aminoglycoside group versus 40% in the unexposed control group.

### Assessment of publication biases

**Fig 3:**
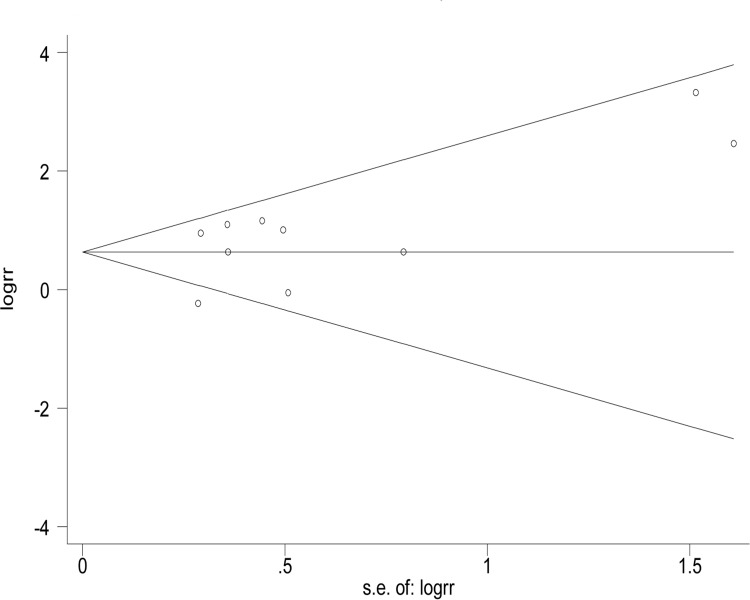


**Fig 4:**
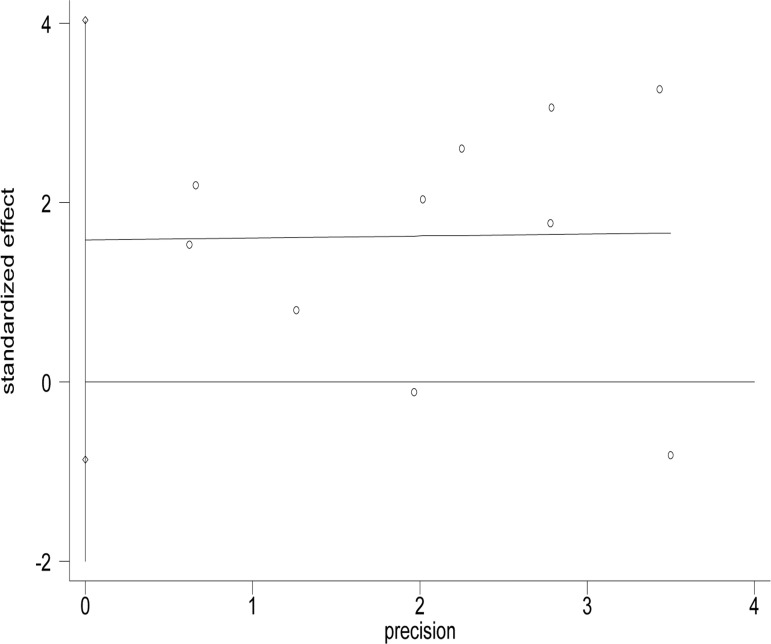


Funnel plots were used to estimate the publication bias. As shown in Figs 3 and 4, no significant asymmetry was found in the funnel plots. Egger’s test (t = 1.49, P = 0.175) and Begg’s test (z = 0.36, P = 0.721) were used to detect publication bias, and there were no significant biases found in the meta-analysis.

## Discussion

The aminoglycosides are broad-spectrum antimicrobials and have rapid bactericidal activity against most Gram-negative aerobic bacteria and staphylococci[11, 25]. They are still commonly used to treat severe bacterial infections in ICUs. Interest in aminoglycosides has been revitalized because of the increasing trend in infections caused by multidrug-resistant bacteria. However, the benefits of these should be weighed up against the adverse effect in aminoglycoside use. Researchers has many concerns about the toxicities of aminoglycosides. Ototoxicity and nephrotoxicity are known safety concerns linked with aminoglycosides therapy. In addition, neuromuscular blockade associated with respiratory depression has also been infrequently reported to be linked with aminoglycosides therapy[26–28]. Aminoglycosides were found to inhibit neuromuscular transmission by inhibiting acetylcholine release from presynaptic nerve terminals[29, 30]. ICUAW is a common neuromuscular complication of critical illness. Patients with ICUAW suffer from longer duration of mechanical ventilation and higher mortality rates. It is essential to evaluate the effect of aminoglycoside use on ICUAW development. After synthesizing the data, this meta-analysis revealed a significant relationship between aminoglycoside use and ICUAW. In addition, the effect of aminoglycoside therapy on ICUAW is complex and may also depend on the cumulative dosage and duration of the aminoglycosides. Of the included studies, the cumulative doses of aminoglycosides were significantly higher in patients with ICUAW than in those without ICUAW in one[17] of the two studies[6, 17], and duration of the aminoglycosides[6, 18] was not found to be a risk factor for ICUAW based on univariate analysis. Additionally, this review should be further viewed in the context of subgroup analysis and the limitations of the included study.

Our subgroup analyses revealed a significant association between the use of aminoglycosides and ICUAW in patients with clinical weakness but not in patients with abnormal electrophysiology. The use of aminoglycosides was found to be an independent risk factor for clinical muscle weakness in the review. ICUAW is essentially a clinically detectable weakness, and clinical examinations were widely used because of its timeliness and convenience. As for some subclinical ICUAW, electrophysiologic studies may have a sensitivity advantage. This may lead to a different outcome.

There are limitations to our review. The limitation of this review was the potential reporting bias resulting from the publication of low-quality and smaller studies with statistically significant associations. Studies with low risk of bias did not demonstrated a significant association between aminoglycoside use and ICUAW, and the negative result was the same for studies limited to relatively large sample sizes. These results partly decreased the stability of the overall effect size. Besides, four included studies did not report whether the assessments were independently blinded for physical therapists or clinicians, which may have further implications on publication and outcome biases. Because few studies were designed to adjust for other independent risk factors and because different risk factors existed across the included studies, we could only perform a univariate primary analysis without adjustment for potential confounders. But in our previous review[31], we included 3 studies that evaluated risk factors using a multivariate approach, and after synthesizing the data, the use of aminoglycosides was also found to be significantly associated with ICUAW (OR 2.27; 95% CI 1.07–4.81). That may partly demonstrate the accuracy of the results of the present review. High levels of heterogeneity were identified for most of the outcomes. Because of lack of reporting and processing the missing data, we could only perform a form of per-protocol analysis. Studies were excluded for the following common reasons: the study design was not a RCT or prospective cohort, insufficient data were reported, and clear diagnostic criteria were lacking. Lacking of RCTs in current studies, only prospective cohort studies were included in the review. This downgraded the quality of evidence and the strength of recommendation.

## Conclusion

The review suggests a statistically significant association between aminoglycoside use and ICUAW; however, this conclusion requires qualification. First, aminoglycosides were more commonly associated with patients with clinical weakness than patients with abnormal electrophysiology. Second, we found that studies with relatively low risk of bias and large sample sizes in our review revealed a small but not a statistically significant increase in developing ICUAW. As a potential risk factor, aminoglycosides still need to be taken into consideration in the future studies on risk factors for ICUAW. And clinicians might be cautious with aminoglycosides, and target limited exposure, shortened administration time and lower total doses of aminoglycosides to reduce the incidence of ICUAW. Additionally, future research should focus on high-quality studies by performing multivariable adjustment for confounders to identify the associations between the use, total doses and duration of aminoglycosides and ICUAW.

## Supporting information

S1 Text PubMed search strategy. (DOCX)

S1 Table Excluded studies and reasons for exclusion. (DOCX)

S2 Table PRISMA checklist. (DOC)
