# Supplementary material for: Aminoglycoside use and intensive care unit-acquired weakness: A systematic review and meta-analysis
Source: PLoS One. 2020 Mar 19;15(3):e0230181. doi: 10.1371/journal.pone.0230181 (PMC7082020; doi:10.1371/journal.pone.0230181)
Supplement: S1 Text — (DOCX) [file pone.0230181.s001.docx]

**Search Strategy PubMed**

**Domain: ICU patients**

(((((((((((critical care) OR ICU) OR intensive care) OR critical ill) OR critical illness) OR critically ill)) OR ((("Intensive Care Units"[Mesh]) OR "Critical Care"[Mesh]) OR "Critical Illness"[Mesh])))

**Determinant：Aminoglycoside**

(("Aminoglycosides"[Mesh]) OR Aminoglycosides) OR Aminoglycoside

**Outcome: Weakness**

((((((((((((("Muscular Diseases"[Mesh]) OR "Muscle Weakness"[Mesh]) OR "Polyneuropathies"[Mesh]) OR "Paralysis"[Mesh]) OR "Paresis"[Mesh]) OR "Neuromuscular Diseases"[Mesh])) OR ((((((((((((((((((paralysis) OR paresis) OR quadriplegia) OR weakness) OR muscular disease) OR muscular diseases) OR neuromuscular disease) OR neuromuscular diseases) OR myopathy) OR myopathies) OR neuropathy) OR neuropathies) OR polyneuropathy) OR polyneuropathies) OR polyneuromyopathy) OR polyneuromyopathies) OR neuromyopathy) OR neuromyopathies)) OR (((((((CIM) OR CIP) OR CIPM) OR CIPNM) OR ICUAW)) OR ICUAP))) OR CINMA))
