# Supplementary material for: Aminoglycoside use and intensive care unit-acquired weakness: A systematic review and meta-analysis
Source: PLoS One. 2020 Mar 19;15(3):e0230181. doi: 10.1371/journal.pone.0230181 (PMC7082020; doi:10.1371/journal.pone.0230181)
Supplement: S1 Table — (DOCX) [file pone.0230181.s002.docx]

| **S2 Table. Excluded studies and reasons for exclusion** | |
| --- | --- |
| **Studies** | **Justification** |
| Abdelmalik (2017) | Retrospective cohort |
| Penuelas (2016) | Failed to meet diagnostic criteria of ICUAW |
| Gupta (2016) | No reported data on aminoglycosides |
| Nguyen (2015) | No reported data on aminoglyscosides |
| Segers (2014) | Study of therapeutic intervention to minimize neuromuscular dysfunction |
| Patel (2014) | No reported data on aminoglyscosides |
| Needham (2014) | Post-hospital outcome study |
| Hermans (2014) | No reported data on aminoglyscosides |
| Fan (2014) | No reported data on aminoglyscosides |
| Al-Dorzi (2014) | Failed to meet diagnostic criteria of ICUAW |
| Parsons (2013) | No reported data on aminoglyscosides |
| Hermans (2013) | No reported data on aminoglyscosides |
| Sharshar (2010) | No reported data on aminoglyscosides |
| Brunello (2010) | No reported data on aminoglyscosides |
| Weber-Carstens (2009) | Data previously included in included study Weber-Carstens 2010 |
| Hermans (2009) | Retrospective cohort |
| Schweickert (2009) | No reported data on aminoglyscosides |
| Sharshar (2009) | Data previously included in included study De Jonghe, 2007 |
| Ali (2008) | No reported data on aminoglyscosides |
| Lefaucheur (2006) | No reported data on aminoglyscosides |
| Khan (2006) | No reported data on aminoglyscosides |
| Van den Berghe (2005) | Insufficient data reported for aminoglyscosides |
| Garnacho-Montero (2005) | No reported data on aminoglyscosides |
| Bercker (2005) | Retrospective cohort |
| Bednarik (2005) | No reported data on aminoglyscosides |
| Druschky (2001) | Failed to meet diagnostic criteria of ICUAW |
| Thiele (2000) | No reported data on aminoglyscosides |
| Tepper (2000) | Case series |
| Miro (1999) | Retrospective cohort |
| Behbehani (1999) | Retrospective cohort |
| Zifko (1998) | Insufficient data reported for aminoglyscosides and ICUAW |
| Coakley (1998) | No reported data on aminoglyscosides |
| Campellone (1998) | No reported data on aminoglyscosides |
| Thiele (1997) | Case-control study |
| Rudis (1997) | Includes therapeutic electrical muscle stimulation |
| Rudis (1996) | Case-control study |
| Leijten (1996) | No reported data on aminoglyscosides |
| Leatherman (1996) | Retrospective cohort |
| Latronico (1996) | All patients with has outcome of interest |
| Leijten (1995) | Insufficient data reported for aminoglyscosides and ICUAW |
| Verheul (1994) | Failed to meet diagnostic criteria of ICUAW |
| Douglass (1992) | No reported data on aminoglyscosides |
| Witt (1991) | Retrospective cohort |
| Zochodne (1987) | Retrospective cohort |
| Abbreviations: ICUAW – intensive care unit acquired weakness | |
